# What to Know about Antimicrobial Coatings in Arthroplasty: A Narrative Review

**DOI:** 10.3390/medicina60040574

**Published:** 2024-03-30

**Authors:** Francesco Onorato, Virginia Masoni, Luca Gagliardi, Luca Costanzo Comba, Fabrizio Rivera

**Affiliations:** 1 Department of Orthopedics and Traumatology, University of Turin, Via Zuretti, 29, 10126 Turin, Italy; francesco.onorato@unito.it (F.O.); virginia.masoni@unito.it (V.M.); lucagglrd@gmail.com (L.G.); 2 Department of Orthopedics and Traumatology, Ospedale SS Annunziata, ASL CN1, Via Ospedali, 9, 12038 Savigliano, Italy; luca.comba@hotmail.com

**Keywords:** periprosthetic joint infections, arthroplasty, antimicrobial coatings, clinical practice, narrative review

## Abstract

Periprosthetic joint infections (PJIs) are one of the most worrying complications orthopedic surgeons could face; thus, methods to prevent them are evolving. Apart from systemic antibiotics, targeted strategies such as local antimicrobial coatings applied to prosthetics have been introduced. This narrative review aims to provide an overview of the main antimicrobial coatings available in arthroplasty orthopedic surgery practice. The search was performed on the PubMed, Web of Science, SCOPUS, and EMBASE databases, focusing on antimicrobial-coated devices used in clinical practice in the arthroplasty world. While silver technology has been widely adopted in the prosthetic oncological field with favorable outcomes, recently, silver associated with hydroxyapatite for cementless fixation, antibiotic-loaded hydrogel coatings, and iodine coatings have all been employed with promising protective results against PJIs. However, challenges persist, with each material having strengths and weaknesses under investigation. Therefore, this narrative review emphasizes that further clinical studies are needed to understand whether antimicrobial coatings can truly revolutionize the field of PJIs.

## 1. Introduction

Periprosthetic joint infections (PJIs) are one of the most challenging complications in orthopedic replacement surgery, leading to adverse medical and healthcare outcomes [1,2,3]. Indeed, they often result in implant failure [1,4], with Bozic et al. reporting infection as the most common cause of total knee arthroplasty (TKA) revision [5] and the third most common cause for hip revision following instability and aseptic loosening, not related to infective causes [6]. According to the literature, PJIs vary up to 2–3% in primary implantation [3,7,8], and their percentage steeply increases with a rate up to more than 50% following large bone defects in revision or tumor surgery [8]. What emerged from the current evidence to prevent PJIs appears to be the avoidance of bacterial adhesion and, thus, biofilm formation [9,10].

Biofilms are composed of extracellular polymeric substances, such as polysaccharides and proteins, intermingled with bacteria, which interact in a complex mechanism [10]. They develop through a cycle where, in the initial phase, bacterial adhesion is usually reversible, evolving into a chronic status where bacteria constantly reproduce [10]. What makes biofilms worrisome is the difficulty of their eradication, since they make it difficult for both the host immune system and drugs to penetrate [10]. The sensitivity of bacteria in biofilms declines since they acquire resistance to antibacterial substances, and their environment is difficult to penetrate due to the poor permeability given by polymeric substances and often because of their acidic and anaerobic conditions [10].

In the past, Gristina introduced the “race for the surface” concept, where bacteria and host cells compete to colonize the implant surface first, avoiding and lowering the probability of the other category of cells adhering to the implant [11]. However, this model was not precise in predicting PJIs in all middle-way situations, where incomplete coverage by a cell category was present and other groups, therefore, partially adhered [12].

For this reason, both surgeons and companies have moved their focus to prosthetic material coatings [9,10] with the aim, as reported by Chen et al., of balancing antibacterial activity with the biocompatibility of the devices at the same time [10].

Additionally, from a medical point of view, patient selection before arthroplasty is fundamental since there are well-reported patient-related risk factors for PJIs, such as diabetes mellitus (DM) and rheumatoid arthritis (RA), in the literature [3,7]. In this high-risk population, it is important not only to comprehensively counsel patients on the potential risks but also to implement the use of the most appropriate antimicrobial strategies to avoid PJIs [1,3,7,13]. Recently, many efforts have been made to improve modifiable patient risk factors [3,7]. In this setting, the concomitant use of an antimicrobial coating could increase the benefits for the most vulnerable patients [1,3,7,13].

Crucial advancements in material technologies and new antimicrobial agents are emerging from basic science studying the physical and chemical properties of coatings, which could be subsequently applied in the field [10]. Indeed, most of the literature currently available depicts the efficacy of antimicrobial coatings either in vitro or on animals [10], while there is a paucity of studies evaluating the outcomes in vivo on humans, with a growing knowledge especially in recent years [9]. 

This narrative review aims to describe antimicrobial coatings, focusing on their employment in arthroplasty orthopedic surgery. The impacts of PJIs in clinical practice are depicted. Patients at risk for whom these implants could be used are presented. Not all the existing coatings are proposed, but rather the ones available and utilized in clinical practice. This will provide orthopedic surgeons with an overview of the most appropriate prosthetic devices for each patient.

### 1.1. Impact of Infections: Quality of Life, Economic Burden, Main Pathogens, and Surgical Strategies

PJIs negatively impact the health of the patient, leading to a lower quality of life (QoL) and prolonged disability [1,14]. As reported by Wildeman et al., both patient-reported outcome measures (PROMs) evaluated through the Euro-QoL-5D-5L and hip function were worst in hip PJIs, with patients requiring ambulatory aid and losing their independence in daily activities, especially after multiple surgical procedures [14]. Moreover, the literature agrees that PJIs are associated with higher mortality, with up to a fivefold increase in the risk of death following revisions for infections with respect to reoperation for aseptic conditions [4,14]. Beyond the negative clinical outcomes, PJIs are a source of massive cost for healthcare systems, with estimated annual hospital charges for hip and knee in the US expected to reach around USD 1.85 billion by 2030 [15]. In addition, microbial infections after prosthetic interventions are becoming more often the subject of legal disputes [16]. 

Evidence-based standards in surgical rooms, such as limiting personnel traffic in the operating theater, have been established, as have perioperative strategies to prevent PJIs [17]. 

The main pathogens associated with PJIs are gram-positive cocci, such as Staphylococcus Aureus and Staphylococcus Epidermidis, either methicillin-sensitive (MSSA, MSSE) or -resistant (MRSA, MRSE), respectively [1,9,18]. Gram-negative bacteria are other isolated organisms [1,18]. Bacteria adopt a set of efficacious survival strategies through the expression of a wide range of specific surface adhesion molecules, which allow them to adhere to and survive on surfaces [10]. Interestingly, what has been reported by Lora-Tamayo et al. concerning hip arthroplasty is that infected total hip arthroplasty (THA) and hip hemiarthroplasty (HHA) have different etiologies and prognoses [19], with Teterycz et al. suggesting that MRSA detection in infected implants is associated with a worse overall outcome with respect to other Staphylococci [20]. However, what the literature agrees on is that the key element of PJIs, at the base of the difficulty in their eradication, arises from the formation of biofilms [9,10,18]. A high bacterial load could overcome local tissue flora, alter immunomodulation, and accelerate biofilm formation [10,18]. Biofilms produced on prosthetic implants are impermeable to the host’s immune response, and antibiotics allow bacteria to acquire multiple drug resistances, too [10,18]. Along with systemic antibiotic administration, several strategies, such as debridement, antibiotics, and implant retention (DAIR), as well as one- or two-stage revision surgery, have been adopted according to the timeline of infection occurrence and the characteristics of the patients and the pathogens [21,22]. For example, Longo et al. reported DAIR to have an overall success rate of 55.5% to 90% for early post-operative and acute hematogenous PJIs in hip and knee prostheses [22].

### 1.2. Patient-Related Risk Factors for Infections

Several patient risk factors have been related to PJIs [1,3,7,10,13]. Apart from DM and RA, already mentioned above and for which there is a strong relationship with PJIs [1,3,7,10,13], a recent meta-analysis of 40 studies has highlighted that Body Mass Index (BMI), cardiovascular diseases (CVDs), chronic pulmonary disease (CPD), neurological disease, immunosuppressive and opioid therapy, and iron deficiency anemia are associated with PJIs [7]. Concerning patients with diabetes, Cancienne et al. suggested a glycated hemoglobin value (HbA1c) of 7.5 mg/dL as a threshold for predicting PJIs following primary THA [23]. The same concept applies to BMI, for which a worse morbid obesity is associated with a more elevated likelihood of PJIs [7]. In this regard, the use of visceral fat measured through computed tomography instead of BMI has recently emerged as a tool for surgeons to assess PJI risk [24]. Age shows a conflicting role in being a factor predisposing to or protecting against PJIs, while males seem to be more vulnerable to infections, as reported by Ren et al. [7]. The use of warfarin and a greater international normalized ratio (INR) were more prevalent among patients developing PJIs [1,25,26]. Interestingly, Tan et al. recently developed a preoperative risk calculator for PJIs, enabling surgeons to identify high-risk individuals who may benefit from additional preventative measures [27].

## 2. Materials and Methods

A descriptive narrative review of the current literature involving antimicrobial coatings in the arthroplasty orthopedic field was conducted. The search was performed on the PubMed, Web of Science, SCOPUS, and EMBASE databases. The search terms were combined using Boolean operators. The keywords considered were “arthroplasty”, “antimicrobial coatings”, “PJI”, “infections”, “silver”, “materials”, and “prosthesis”. Both full names and abbreviations were searched. Updated studies with all levels of evidence, independently from their design, were included. No time limitation was applied, but the most recent literature, especially dating from 2018 to 2024, was analyzed, with some exceptions concerning milestone articles. No language limitation was considered. Of utmost importance, since the main focus of this paper was to provide an insight into prosthetic clinical practice along with a summary of the possible solutions an orthopedic surgeon could adopt, referrals were mainly to human studies. For this reason, even though in the literature many recent in vitro and animal studies have been reported [10], some preclinical studies have not been mentioned on purpose. Nonetheless, since clinical human studies are limited, especially concerning the arthroplasty domain, coatings used in other clinical settings, such as trauma, have been mentioned to illustrate their future potential role in the arthroplasty field as well. In the literature, there is no evidence yet of a universally accepted classification of coating technologies. For this reason, three main categories based on their use in clinical practice have been described to simplify the description. The first section is about silver, which deserves a specific paragraph due to its large-scale use in clinical oncological prosthetic settings. The section about antibiotic coatings deals with their use in addition to both bone cement and implant surfaces, either directly or loaded into hydrogel. The last section concerns “other coatings”, referring either to already-adopted materials that have had less success in clinical practice or to innovative materials that have emerged in recent years. 

## 3. Antimicrobial Coating Alternatives

### 3.1. Silver Coating

Silver (Ag) is one of the most commonly employed metals in orthopedics to decrease PJIs due to its antimicrobial properties [8,10,28,29]. It has been highly adopted in the oncological field and revision world, where megaprosthesis implantation prevails [8], and has shown potential efficacy in reducing PJIs [8,30]. Donati et al., when comparing silver-coated with titan-uncoated hip megaprostheses, reported a protective effect of silver, especially in the first months after surgery [30]. Furthermore, Streitbuerger et al. recently described not only the decreased infection rate with a silver-coated proximal femoral replacement but also the reduced necessity for two-stage procedures, with minor surgeries such as DAIR being employed in silver implants [31]. Apart from the oncological field, silver-coated megaprostheses have been adopted in trauma patients after infection as revision surgery, for example, by Hussmann et al. [32]. Even if the study was interrupted due to the increase in silver serum levels and ethical concerns, Massè et al. investigated the effect of silver-coated pins in external fixators [33]. Considering other clinical applications, silver has been added to poly-methyl methacrylate (PMMA) in hip spacers [34], and Wilding et al. proposed a silver-coated intramedullary nail for knee arthrodesis instead of limb amputation as a solution for patients with septic revised TKA [35]. Modern silver-coated implants have been introduced in spinal surgery too, with favorable outcomes, as reported by Morimoto et al., who described the use of a silver-containing hydroxyapatite (Ag-HA)-coated cage for posterior lumbar interbody fusion [13]. The antimicrobial properties of silver have been deeply studied [8,10,28,29]. It works through several mechanisms of action since it interrupts the bacterial metabolic cycle by blocking the respiratory chain and destroying cell walls; it interferes with cell translation and transcription processes, and it also induces reactive oxygen species (ROS) [8,10,28,29]. For these reasons, it has a broad spectrum of activity against both gram-positive and gram-negative bacteria, protozoa, viruses, and fungi, with a reported low risk of developing resistance [8,10,28,29]. What has been recently introduced in clinical practice is the use of nanoparticles (NPs) [8,10,28,29,36]. Silver nanoparticles (AgNPs) have been shown to be more efficacious against bacteria and to pose the least risk of resistance with respect to direct silver ion release [8,10,28,29,36]. Indeed, bacterial resistance to AgNPs has not been reported yet [8,36]. This potential of AgNPs has been described to be due to the greater surface area for ion discharge, a more controlled silver release, and a low chance of forming complexes with serum proteins [8,10,28,29,36,37]. AgNPs have also been investigated in combination with antibiotics or antimicrobial coatings, showing greater antibacterial properties and a synergistic effect [36]. However, even AgNPs are not free from risks, and particle size has been reported to be more important than concentration or dose, with smaller AgNPs being more toxic due to the higher surface/volume ratio leading to higher oxidation and dissolution [36]. Another issue questioned is the duration of silver antibacterial activity since a surface degradation process usually ensues [8,10,28,30]. Donati et al., in their scanning electron microscopy (SEM) analysis of retrieved implants at 18 and 27 months, showed wear of the coating, with silver particles almost depleted [30]. Despite this, what the literature investigates and what especially concerns surgeons in clinical practice are silver drawbacks [8,10,28,29,38]. Both systemic and local side effects have been documented with high silver concentrations [8,10,28,29,38]. Regarding systemic consequences, cases of nephrotoxicity, hepatopathy, leukopenia, and peripheral neuropathies have been cited [8,38,39]. 

In 1985, Vik et al. published a case report in The Lancet of severe muscle paralysis following the implantation of silver-containing bone cement in hip revision arthroplasty [40]. Trop et al. described liver dysfunction in a burn patient treated with silver-coated dressings [41].

Nonetheless, as reported by Li et al. [38] as well as Fiore et al. [8], the blood silver concentrations with modern implants recorded in the literature are far from the threshold of silver poisoning, and no major systemic side effects have been reported recently. In agreement, Smolle et al. documented no systemic effects in their patient’s cohort in 2022, but they proposed rigorous monitoring of silver concentrations in patients with these material-containing implants [42]. Concerning local effects, they can be split into two main parts [8,10,28,29,38,39]. The first one is argyria, which consists of a local blue-grayish skin discoloration due to silver accumulation [8,28,38,39]. In this regard, as documented by Li et al. [38] and Fiore et al. [8], percentages of argyria vary in the literature, but the ones documented usually involve implants with a higher silver amount. Recently, Smolle et al. reported four cases of argyria with an incidence of 8.7% and no associated systemic effect [42]. Contrarily, Kawano et al. did not report the argyria phenomenon [39]. The other local aspect of utmost importance influencing clinical practice is the concern about the osteointegration of silver-coated implants [8,10,28,29,39]. Chen et al. [10] and other authors [8,28,29] raised apprehensions about bony metabolism around this metal. Diez-Escudero et al., in analyzing the function of silver in the arthroplasty world, illustrated how the use of silver on implants planned for cementless fixation is unusual, with a balance between osteoconductivity and antibacterial properties of paramount priority [28]. Indeed, all the megaprostheses described have silver either on non-articulating surfaces or on facets not directly in contact with the cement or the host bone [8,28]. Recently, only one model of prosthesis has been described, presenting a combination of hydroxyapatite (HA) and silver oxide (Ag) planned for cementless fixation [28,39]. In this regard, Kawano et al., by removing two Ag-HA acetabular components in hip revision for recurrent dislocation, described good bone ongrowth. They observed white osseous tissue, both detected macroscopically and confirmed by SEM, on the retrieved implants [43]. Moreover, the same authors [39] published the results of a cohort of fifty cases with a five-year follow-up in which a Ag-HA prosthesis was implanted, reporting good clinical outcomes and no radiographic failures, further supporting the recent development of Ag-HA-coated implants intended for cementless fixation [13,39,44]. Finally, economic burden should be considered [8,28]. Silver megaprostheses are more expensive than non-silver-coated implants, but as reported by Fiore et al., with a reduction in peri-implant infections, there could be a paradoxical cost-saving effect [8].

### 3.2. Antibiotic Coatings

Systemic antibiotics are one of the frontline tools against infection, but they are not without drawbacks; first, their concentration at the target area could be limited due to anatomical tissue barriers, and second, they could provoke organ toxicity [9,10,45]. For this reason, antibiotic coatings for local delivery have been employed in clinical practice; however, the literature agrees on unsolved questions about their kinetics and pharmacodynamics [9,10,45]. Moreover, the main issue related to antibiotics is the emergence of antibiotic resistance [9,10]. A study conducted by Anagnostakos and Sahan showed that in patients with PJIs undergoing hip or knee cement spacers or beads, of all the organisms isolated, 54.2% were resistant to clindamycin, whereas 37.1% were resistant to gentamicin [46]. Several local antibiotic formulations exist [9,10,47]; for example, Singh et al. sprayed vancomycin powder directly into the surgical site at the time of definitive fixation in high-energy pilon and tibial plateau fractures, despite this procedure not being effective in preventing deep infections [48]. Antibiotics were added to bone cement, too, either in primary prosthetic implantation, in the case of joint spacers, or in beads [10,46,49,50]. What has emerged in the literature is the use of antibiotics coating the surface of orthopedic implants such as nails and prosthetic devices [9,10,45,47]. In clinical practice, gentamicin is one of the most common antibiotics used, particularly in intramedullary nails [9,47]. Franz et al. demonstrated that the use of gentamicin-coated nails in patients with Gustilo–Anderson (GA) type III open tibial fracture led to a lower infection rate and was cost-saving [51]. In their review, Kalbas et al. support the use of antibiotic-coated nails [52], in line with De Meo et al., who sustained their use in open fractures and nonunion in high-infectious-risk settings [53]. To modify the kinetic properties, antibiotic-loaded hydrogel coatings such as Defensive Antibacterial Coatings (DACs) have been employed in trauma and prosthetic settings [9,10,45,53]. As reported by De Meo et al., hydrogel coatings have benefits and drawbacks [53]. Since they are applied manually directly by the surgeons, they could be laid in any fixation device or prosthesis, and the appropriate antibiotic could be chosen, but the uniform application of materials on the implant is up to the surgeon’s ability [53]. The use of DACs was effective in reducing PJIs by De Meo et al. in the arthroplasty world, where they were applied to prostheses implanted for aseptic revisions [54]. Romanò et al. demonstrated that antibiotic-loaded hydrogel coatings on hip and knee prosthetic implants reduce the rate of early surgical site infections without any detectable side effects [55].

### 3.3. Other Coatings

Several other coatings have been reported mainly in vitro or in animal studies, without strong conclusions in clinical practice [9,10,38,47]. Iodine is a broad-spectrum antiseptic agent that has been added to several orthopedic implants, including megaprostheses and hip and knee prostheses [9,10,38,47]. Savviddou et al., in their meta-analysis, reported only one study utilizing iodine coating without drawing final considerations [9,56]. The study presented by Miwa et al. reported encouraging results for iodine coating devices after malignant bone tumor resections [56]. This agent was employed by Tsuchiya et al. in different titanium orthopedic devices, and it proved to be effective both in the prevention and treatment of infections, with excellent bone ingrowth and ongrowth around the implants [57]. Systemic side effects, such as thyroid toxicity, were not detected [57]. Ongoing research is also directed towards antimicrobial peptide (AMP) coatings [10,58] and chitosan [10,45,59]. This polymer has been added to HA on titanium implants, showing inhibition of bacterial growth in an in vitro study [59]. However, according to current knowledge, no clinical application has been reported in the literature yet [10,45,59]. Similar to Ag, other metals such as copper (Cu) and zinc (Zn) have been proposed [10,38,60,61]. For example, Zn, apart from its bactericidal properties, is an essential element for multiple steps in bone formation, so it could have a promotive function in osteointegration [10]. Instead, Cu could enhance osteogenesis [10]. However, despite their potential antimicrobial activities, there are concerns regarding their side effects, and, therefore, both elements are still under investigation [10,38,60,61]. Of interest, Magnesium (Mg) has been added as a coating to porous titanium implants in orthopedics in vitro and in animal studies, revealing enhanced osteogenesis and, at the same time, antimicrobial properties [10,45,62,63]. Last, what has recently emerged from the literature is the employment of a titanium nail coated with a noble alloy made of gold, silver, and palladium [64,65]. This alloy, acting through a galvanic mechanism, already exhibited antimicrobial properties when applied to Foley catheters to prevent urinary tract infections in a large multicenter clinical study [66]. Concerning orthopedic settings, Karupiah et al. used a metal alloy-coated titanium nail in the management of GA type IIIa or IIIb femoral or tibial fractures, attaining bone union and preventing infections [64]. Kotsarinis et al. used metal alloy-coated titanium nails in the treatment of tibial shaft fractures in high-infectious-risk settings, with encouraging results [65].

### 3.4. Future Directions, Limitations, and Challenges

With joint replacement being one of the most requested and successful procedures worldwide, preventing complications such as PJIs is essential [2,3,4]. Despite the introduction of antimicrobial coatings, challenges persist [2,3,4]. Silver, as well as antibiotic coatings and other materials, have been proposed, and all of them have shown some strengths and weaknesses that need further understanding and investigation. However, promising results in clinical practice are expected since, as reported by Savvidou et al. in a recent meta-analysis, patients undergoing surgery with antimicrobial-coated implants presented a decrease in PJI rates [9]. Complications related to organ side effects, as well as the feared lack of biocompatibility and osteointegration of the devices with cementless fixation, appear to be improving based on the literature. For example, the Ag-HA association has led to good results [39]. However, there is still a paucity of human clinical studies, often with heterogeneity in the materials employed. In this regard, Fiore et al., in a recent meta-analysis, described the three main silver-coated megaprostheses available and their different characteristics, such as the amount of silver content, the coating composition, and the production technology [8]. The same is true for other newly discovered coatings, where different assortments in preparation and composition are presented [10]. In addition to the heterogeneity of the coating technologies, there is a lack of a universally accepted classification [10,67]. Among the ones most commonly used, a subdivision based on the strategy of action has been developed [67]. This classification categorizes antibacterial coatings into three groups: passive surface-finishing/modification agents, active surface-finishing/modification agents, and perioperative antibacterial local carriers or coatings [67]. Concerning antibiotics, doubts remain, as reported by Anagnostakos and Sahan, about their correct choice in accordance with the susceptibility of microorganisms, since bacteria are not always identified preoperatively, and when identified intraoperatively, samples could show additional bacteria [46]. Moreover, as reported by Bistolfi et al. in their review, the use of antibiotic-loaded cement in primary arthroplasty could evolve into antibiotic resistance, with its use suggested only for primary implants in high-risk patients or revision procedures [49]. While engineers and biochemists believe they could develop effective, durable, and safe coatings preventing bacterial adhesion and the formation of biofilm on surfaces, the potential detrimental side effects and the costs still remain a major issue for surgeons, clinicians, and healthcare systems. A close collaboration between the parties could certainly improve these still-unsolved issues. Therefore, there is a big urge to supplement the literature with more studies, such as RCTs, employing more homogenous and reproducible inclusion criteria and patient selection to understand whether antimicrobial coatings can truly revolutionize the field of PJIs.

## 4. Conclusions

PJIs are still one of the most daunting complications orthopedic surgeons could face, often necessitating multiple surgeries, leading to lower QoL, and strongly impacting healthcare systems. For these reasons, several targeted strategies have been developed in arthroplasty aimed at minimizing systemic side effects, mostly through local antimicrobial coatings. With silver already being amply utilized in the oncological field with successful outcomes, other coatings such as silver associated with hydroxyapatite for cementless fixation, antibiotic coatings, antibiotic-loaded hydrogel coatings, and other materials have been utilized in clinical arthroplasty settings with promising results, especially when applied to the appropriate patients.

## Data Availability

All the studies cited are listed in the reference list.
